# Fucoidan attenuates metabolic dysfunction-associated fatty liver disease in mice and is associated with gut microbiota remodeling and AMPK/PPARγ signaling

**DOI:** 10.3389/fmicb.2026.1936901

**Published:** 2026-10-27

**Authors:** Yukun Li, Mengchen Shan, Qinxin Liu, Xue Jing, Zan Wang, Tao Mao, Xingsi Qi, Bin Cao, Zibin Tian, Rongshuang Han

**Affiliations:** 1 Department of Gastroenterology, The Affiliated Hospital of Qingdao University, Qingdao, China; 2 Department of Gastroenterology, Qingdao Cardiovascular Hospital, Qingdao, China

**Keywords:** fucoidan, gut microbiota, gut–liver axis, MAFLD, AMPK/PPARγ signaling

## Abstract

**Background:**

Metabolic dysfunction-associated fatty liver disease (MAFLD) is a growing metabolic disorder closely linked to gut micro biota dysbiosis and impaired gut–liver communication. Dietary interventions targeting the gut micro biota represent a promising strategy for MAFLD management. Fucoidan (FUC), a marine-derived bioactive polysaccharide with limited host digestibility, is primarily metabolized by intestinal microorganisms. However, whether FUC protects against MAFLD in association with micro biota-mediated mechanisms remains incompletely understood.

**Methods:**

C57BL/6J mice were fed a high-fat diet (HFD) for 14 weeks, while the FUC intervention group received FUC by oral gavage at a dose of 200 mg/kg/day over the same period. Body weight, adiposity, serum lipid and glucose metabolism, liver function, and histopathology were evaluated. Gut micro biota composition was analyzed by 16S rRNA sequencing, and serum metabolomic profiling was performed using untargeted metabolomics combined with KEGG enrichment and Spearman correlation analyses.

**Results:**

FUC supplementation markedly attenuated HFD-induced obesity, impaired glucose tolerance, dyslipidemia, hepatic steatosis, and HFD-associated alterations in intestinal barrier-related structural and molecular features. Microbiome analysis revealed that FUC administration was associated with changes in gut microbial diversity and community composition, including altered relative abundances of *Akkermansia, Adlercreutzia*, and *Dubosiella*. Metabolomic analysis further demonstrated changes in systemic metabolic profiles, particularly tricarboxylic acid (TCA) cycle-related metabolites. Correlation analyses identified associations among microbial features, circulating metabolites, and MAFLD-related phenotypes. FUC administration was associated with gut micro biota remodeling and altered hepatic AMPK/PPARγ signaling, accompanied by improved metabolic homeostasis and reduced hepatic inflammation.

**Conclusion:**

Our findings demonstrate that FUC alleviates HFD-induced MAFLD and is associated with remodeling of the gut micro biota, alterations in circulating TCA cycle-related metabolites, and changes in hepatic AMPK/PPARγ signaling. Integrated analyses suggest a potential gut micro biota–metabolism–liver regulatory network associated with the hepato protective effects of FUC. These findings provide potential mechanistic insights into the beneficial effects of FUC and support further investigation of marine-derived polysaccharides as dietary strategies for metabolic disease management.

## 1 Introduction

Metabolic dysfunction-associated fatty liver disease (MAFLD) has emerged as the most prevalent chronic liver disease worldwide, affecting more than 30% of the global population, with its prevalence continuingto rise (Argenziano et al., 2024). MAFLD is closely associated with obesity, type 2 diabetes mellitus, insulin resistance, and cardiovascular disease (Cao et al., 2024; Zhao et al., 2024), and can progressively develop into metabolic dysfunction-associated steatohepatitis (MASH), liver fibrosis, cirrhosis, and even hepatocellular carcinoma, thereby imposing a substantial global health burden (Crane et al., 2023; Pipitone et al., 2023). MAFLD emphasizes the central role of metabolic risk factors in the development and progression of fatty liver disease (Abdelhameed et al., 2024; Fouad et al., 2024). In recent years, the terminology has gradually evolved toward metabolic dysfunction-associated steatotic liver disease (MASLD), reflecting an updated framework for the classification of steatotic liver disease. In the present study, we use the term MAFLD to describe the disease phenotype and experimental model because the study was designed and interpreted within the metabolic dysfunction-centered MAFLD framework, and the high-fat diet-induced mouse model does not directly correspond to the current clinical diagnostic criteria for MASLD.

A high-fat diet (HFD) is one of the major contributors to MAFLD development (De Cól et al., 2024). Chronic HFD consumption promotes excessive hepatic lipid accumulation, impairs fatty acid β-oxidation, and induces oxidative stress, ultimately leading to hepatic steatosis and inflammatory injury (Kuchay et al., 2020). Despite the rapidly increasing prevalence of MAFLD, effective pharmacological therapies remain limited, and current therapeutic strategies primarily focus on restoring lipid homeostasis and enhancing fatty acid oxidation (Khaznadar et al., 2024; Rojas et al., 2022). Among the key regulators of lipid metabolism, the peroxisome proliferator-activated receptor (PPAR) family, comprising PPARα, PPARβ/δ, and PPARγ, functions as ligand-activated nuclear receptors that are widely expressed in the liver, adipose tissue, and intestine (Monroy-Ramirez et al., 2021; Peiseler et al., 2022). PPARγ not only regulates adipocyte differentiation but also maintains lipid homeostasis by controlling the transcription of genes involved in lipid metabolism, making it an attractive therapeutic target for MAFLD (Cai et al., 2025; Szántó et al., 2021).

AMP-activated protein kinase (AMPK), a master cellular energy sensor, plays a pivotal role in maintaining energy homeostasis by coordinating lipid synthesis and fatty acid oxidation (Steinberg and Hardie, 2023). AMPK activation suppresses lipogenic pathways while promoting PPAR-mediated fatty acid β-oxidation, thereby reducing hepatic lipid deposition and improving metabolic homeostasis (Shi et al., 2023; Steinberg et al., 2025). Accumulating evidence has demonstrated that the AMPK/PPAR signaling axis is critically involved in the pathogenesis of MAFLD and represents a promising target for therapeutic intervention (Fang et al., 2022).

Fucoidan (FUC), a naturally occurring sulfated polysaccharide primarily composed of α-L-fucose and derived from brown seaweeds (Zahariev et al., 2023), has attracted considerable attention because of its diverse biological activities, including anti-inflammatory, antioxidant, lipid-lowering, glucose-regulating, and anti-obesity effects (Li et al., 2020). Owing to its limited digestibility by host enzymes, FUC is likely to interact extensively with the gut micro biota, which has been implicated in its biological activities. Previous studies have shown that FUC can reshape the gut microbial community, promote the growth of selected bacterial taxa, suppress intestinal and systemic inflammation, and ameliorate metabolic disorders in association with gut–liver axis alterations (Sun et al., 2025). However, the potential involvement of AMPK/PPARγ signaling in the hepato protective effects of FUC and its relationship with FUC-associated gut micro biota remodeling remain insufficiently characterized.

In the present study, we investigated the protective effects of FUC against MAFLD and explored its potential associations with gut micro biota and host metabolic signaling. Using an HFD-induced MAFLD mouse model, we integrated gut micro biota profiling and serum metabolomics to characterize changes in microbial composition, systemic metabolic profiles, and hepatic signaling responses following FUC administration. We found that FUC alleviated hepatic steatosis and metabolic dysfunction, improved intestinal barrier-associated features, remodeled gut microbial composition, and was associated with altered hepatic AMPK/PPARγ signaling and TCA cycle-related metabolic profiles. Integrated analyses further identified potential associations among gut micro biota, circulating metabolites, and host metabolic phenotypes. These findings provide potential mechanistic insights into the hepato protective effects of FUC and highlight a potential gut micro biota–metabolism–liver regulatory network that warrants further causal investigation.

## 2 Materials and methods

### 2.1 Reagents

The fucoidan (FUC; purity ≥90%) used in this study was a commercial product supplied by Bright Moon Seaweed Group Co., Ltd. (Qingdao, Shandong, China), which was industrially extracted and purified from *Saccharina japonica*. Its relative molecular mass was determined to be 83.5 kDa by Gel Permeation Chromatography with a refractive index detector (GPC-RID). Briefly, the seaweed sample was sliced, minced, and extracted with 10 volumes of deionized water at 70 °C for 12 h. After centrifugation, alginate was removed by calcium chloride precipitation, and FUC was subsequently precipitated from the supernatant with five volumes of absolute ethanol. The precipitate was lyophilized and stored at 4 °C until further use.

### 2.2 Animal experiments

Eighteen six-week-old male C57BL/6J mice were obtained from Vital River Laboratory Animal Technology Co., Ltd. (Beijing, China). Animals were housed under specific pathogen-free (SPF) conditions at 22 ± 2 °C with a relative humidity of 50–60% under a 12-h light/dark cycle, with *ad libitum* access to food and water. All experimental procedures were approved by the Ethics Committee of the Affiliated Hospital of Qingdao University (AHQU-MAL20241225HRS).

Following a one-week acclimatization period, mice were randomly assigned to three groups (*n* = 6 per group): Control group (CON), High-fat diet (HFD) group, High-fat diet+FUC (200 mg/kg) group. Three mice were housed per cage, with animals from each experimental group distributed across different cages to minimize potential cage effects. The CON group fed a standard chow diet (Keaoxieli Feed Co., Ltd., Tianjin, China), containing ≥18.0% crude protein, ≥4.0% crude fat, ≤ 5.0% crude fiber, and ≤ 8.0% crude ash, with calcium and total phosphorus contents of 1.0–1.8% and 0.6–1.2%, respectively. The HFD and FUC groups fed a 60 % fat diet (Research Diets, D12492) (Shen et al., 2022). FUC was administered by oral gavage at a dose of 200 mg/kg/day for 14 consecutive weeks (Liu et al., 2022; Sun et al., 2023). During this period, the CON and HFD groups received an equivalent volume of sterile water by oral gavage.

Body weight, food intake, and water consumption were monitored weekly throughout the study. Longitudinal blood biochemical measurements were obtained from the same animals throughout the intervention. Blood samples for longitudinal biochemical measurements were collected from the same animals at the indicated time points using the tail vein, with the sampling volume maintained within the institutionally approved limits. Serum samples were obtained after appropriate clotting and centrifugation and were stored at −80 °C until analysis. At the end of the 14-week intervention, mice were euthanized by CO_2_ inhalation in accordance with the guidelines of the Institutional Animal Care and Use Committee (IACUC). Blood samples were collected immediately after euthanasia. The liver, adipose tissue, and fecal samples were harvested, immediately snap-frozen, and stored at −80 °C until further analysis.

### 2.3 Biochemical analysis

Serum levels of alanine aminotransferase (ALT), aspartate aminotransferase (AST), total cholesterol (TC), and triglycerides (TG) were determined using an automated biochemical analyzer according to the manufacturer's instructions.

An intraperitoneal glucose tolerance test (IPGTT) was performed during week 13 of the intervention. Following an overnight fast, mice received an intraperitoneal injection of glucose (2.0 g/kg body weight; Sigma-Aldrich, G7021). At the end of the 14-week intervention period, random (non-fasting) blood glucose levels were measured using blood collected from the tail vein without prior fasting. Blood glucose levels were measured using a glucometer (Accu-Chek, Roche Diagnostics, Shanghai, China) with blood collected from the tail vein immediately before glucose administration (0 min) and at 15, 30, 60, and 120 min thereafter (Yavorov-Dayliev et al., 2023). The area under the curve (AUC) was calculated according to the formula (de la Garza et al., 2013).

### 2.4 Histological analysis

Hematoxylin and eosin (H&E) staining. Fresh liver and ileal tissues were fixed in 4% paraformaldehyde, dehydrated through a graded ethanol series, embedded in paraffin, and sectioned at a thickness of 4 μm. Tissue sections were stained with H&E according to standard protocols. Histopathological changes, including hepatic steatosis, inflammatory cell infiltration, and colonic morphology, were evaluated under a light microscope.

Oil Red O (ORO) staining. Fresh liver tissues were fixed in 4 % paraformaldehyde for at least 24 h, cryoprotected in 30 % sucrose until fully infiltrated, embedded in optimal cutting temperature (OCT) compound, and cryosectioned at a thickness of 10 μm. Frozen sections were stored at −20 °C before staining with ORO according to the manufacturer's instructions. Hepatic lipid accumulation was assessed by light microscopy.

Alcian blue/periodic acid-Schiff (AB/PAS) staining. Fresh colon tissues were fixed in Carnoy's solution, dehydrated through a graded ethanol series, embedded in paraffin, and sectioned at 4 μm. Sections were stained using the AB/PAS method to evaluate colonic mucus production. Goblet cells were quantified by light microscopy. Goblet cells were counted in multiple crypts from each mouse, and the mean value for each mouse was used for statistical analysis.

Histological scoring was performed by an investigator blinded to group allocation.

### 2.5 Enzyme-linked immunosorbent assay (ELISA)

Liver tissues were homogenized under ice-cold conditions and centrifuged at 1000g for 20 min at 4 °C. The resulting supernatants were collected for subsequent analysis. Hepatic concentrations of tumor necrosis factor-α (TNF-α) and interleukin-6 (IL-6) were quantified using commercially available ELISA kits (Elabscience, China) according to the manufacturer's instructions.

### 2.6 Western blot analysis

Total protein was extracted from liver and colon tissues using radioimmunoprecipitation assay (RIPA) lysis buffer according to the manufacturer's instructions. Protein concentrations were determined using a bicinchoninic acid (BCA) protein assay. Equal amounts of protein (30 μg) were separated by 10% sodium dodecyl sulfate–polyacrylamide gel electrophoresis (SDS-PAGE) and transferred onto 0.45-μm polyvinylidene fluoride (PVDF) membranes (Merck Millipore, USA). Membranes were blocked with 5 % bovine serum albumin (BSA) and incubated overnight at 4 °C with primary antibodies against p-AMPK (2535S; Cell Signaling Technology), AMPK (5831S; Cell Signaling Technology), PPARγ (C26H12; Cell Signaling Technology), Occludin (91131; Cell Signaling Technology), and MUC2 (ab272692; Abcam). After washing, membranes were incubated with the appropriate horseradish peroxidase (HRP)-conjugated secondary antibodies for 1.5 h at room temperature. Protein bands were visualized using an enhanced chemiluminescence (ECL) detection kit (Elabscience, China) and quantified with ImageJ software. Protein expression levels were normalized to GAPDH. Each biological sample was analyzed in technical triplicate.

### 2.7 Reverse transcription-quantitative PCR (RT-qPCR)

Total RNA was extracted from colon tissues using TRIzol reagent according to the manufacturer's instructions (TaKaRa, Japan). RNA concentration and purity were determined prior to reverse transcription. Equal amounts of total RNA were reverse-transcribed into complementary DNA (cDNA) using a reverse transcription kit (Accurate Biology, AG1711, China). RT-qPCR was performed using SYBR Green Master Mix on a real-time PCR detection system. Each reaction was carried out in a final volume of 20 μL under the following cycling conditions: initial denaturation at 95 °C for 5 min, followed by 40 amplification cycles of 95 °C for 10 s and 60 °C for 30 s. All samples were analyzed in triplicate. Gene expression levels were normalized to GAPDH, and relative mRNA expression was calculated using the 2^∧^−ΔΔCt method. The primer sequences used in this study are listed in Table 1.

**Table 1:** Primer sequences used for RT-qPCR in mice intestine tissues.

Gene	Forward primer (5^′^ → 3^′^)	Reverse primer (5^′^ → 3^′^)
*MUC2*	GGGTGGAAGTGGCATTGTGTG	ACAACGAGGTAGGTGCCATCC
*Occludin*	AGCAGCGGTGGTAACTTTGAG	CCTCCAGCTCATCACAGGACTC
*GAPDH*	GTATGACTCCACTCACGGCAAA	GGTCTCGCTCCTGGAAGATG

### 2.8 16S rRNA gene sequencing

16S rRNA gene sequencing was performed by Majorbio Bio-Pharm Technology Co., Ltd. (Shanghai, China). Total microbial DNA was extracted from fecal samples using the FastPure Stool DNA Isolation Kit (Shanghai, China) according to the manufacturer's instructions. The V3–V4 hypervariable region of the bacterial 16S rRNA gene was amplified using the universal primers 338F (5′-ACTCCTACGGGAGGCAGCA-3′) and 806R (5′-GGACTACHVGGGTWTCTAAT-3′). The resulting amplicons were sequenced on an Illumina MiSeq platform. Raw sequencing reads were quality filtered and denoised using DADA2 implemented in QIIME 2 (version 2020.2). The resulting high-quality sequences were resolved into amplicon sequence variants (ASVs) using a denoising procedure. Taxonomic classification of ASVs was performed using the naïve Bayes classifier against the SILVA 16S rRNA database (v138) database. Microbial community analyses were subsequently conducted using the QIIME pipeline, and downstream bio informatic analyses were performed using the Major bio Cloud Platform (https://cloud.majorbio.com/). Alpha diversity was evaluated using the Chao and Shannon diversity indices, and differences among groups were assessed using the Kruskal–Wallis test. Beta diversity was evaluated based on Bray-curtis dissimilarity, and principal coordinates analysis (PCoA) was performed to visualize differences in microbial community structure among samples. Permutational multivariate analysis of variance (PERMANOVA), a non-parametric multivariate statistical test, was performed based on the Bray-curtis dissimilarity matrix to determine whether microbial community structures differed significantly among groups. Differentially abundant bacterial taxa from the phylum to genus levels were identified using linear discriminant analysis effect size (LEfSe), with an LDA score >2.0 and *P* < 0.05 considered indicative of statistically significant differences.

### 2.9 Metabolomic analysis

Serum samples were collected from mice for untargeted metabolomic profiling. 100 μL of serum was mixed with 400 μL of extraction solvent (acetonitrile/methanol, 1:1, v/v) in a 1.5 mL centrifuge tube and vortexed for 30 s. The samples were subjected to low-temperature ultrasonic extraction, followed by centrifugation at 13,000 × g for 15 min at 4 °C. The resulting supernatants were collected and dried, then reconstituted in 100 μL of acetonitrile/water (1:1, v/v). After centrifugation at 13,000 × g for 10 min at 4 °C, the supernatants were collected for LC–MS analysis. An aliquot of 3 μL of each sample was separated on an HSS T3 column (100 mm × 2.1 mm i.d., 1.8 μm). Mobile phase A consisted of water/acetonitrile (95:5, v/v) containing 0.1% formic acid, while mobile phase B consisted of acetonitrile/isopropanol/water (47.5:47.5:5, v/v/v) containing 0.1% formic acid. The flow rate was 0.40 mL/min, and the column temperature was maintained at 40 °C. Mass spectrometric data were acquired in both positive and negative ionization modes. The raw LC–MS data were uploaded to the Major bio Cloud Platform (https://cloud.majorbio.com/) for data processing and statistical analysis. The data matrix was normalized using sum normalization, and variables with a relative standard deviation (RSD) >30% in quality control (QC) samples were removed. The resulting data were subsequently log10-transformed. Principal component analysis (PCA) was performed using the ropls package (version 1.6.2) in R. Differential metabolites were identified based on the variable importance in projection (VIP) values from the OPLS-DA model and Student's *t*-test, with VIP >1.0 and *P* < 0.05 considered statistically significant. Differential metabolites were annotated against the Kyoto Encyclopedia of Genes and Genomes (KEGG) database (https://www.kegg.jp/kegg/pathway.html). Python packages “scipy. stats” was used to perform enrichment analysis to obtain the most relevant biological pathways for experimental treatments.

### 2.10 Statistical analysis

Statistical analyses were performed using GraphPad Prism 9.0 software. For longitudinal measurements repeatedly collected from the same animals, including body weight, serum lipid levels, ALT, AST, and blood glucose, two-way repeated-measures analysis of variance (ANOVA) was performed, with treatment group and time as the two factors. Dunnett's multiple-comparisons test was used for *post hoc* comparisons. For comparisons whereas the Kruskal–Wallis test was applied for non-normally distributed data. Data are presented as mean ± standard deviation (SD). A two-sided *P* < 0.05 was considered statistically significant (Liu et al., 2024). Spearman correlation analysis was performed to evaluate the associations between representative serum metabolites, gut microbial taxa, and metabolic parameters. *P* values from the correlation analyses were adjusted for multiple comparisons using the Benjamini–Hochberg false discovery rate (FDR) procedure. Associations with a Spearman correlation coefficient |*r*| > 0.6 and an FDR-adjusted *P* value (*q* value) < 0.05 were considered statistically significant. among multiple groups at a single time point, one-way ANOVA followed by Tukey's multiple-comparisons test was used for normally distributed data,

## 3 Results

### 3.1 FUC reduces body weight gain and adipose tissue mass in HFD-induced MAFLD mice

To evaluate the effects of FUC on MAFLD, an HFD-induced murine model was established. Male C57BL/6J mice were fed either a normal chow diet or HFD for 14 weeks. The FUC intervention group received FUC by oral gavage at a dose of 200 mg/kg/day over the same period. (Figure 1A). All mice maintained a healthy physiological state during the 14-week intervention. Compared with the CON group, HFD-fed mice exhibited a progressive increase in body weight, whereas FUC administration significantly attenuated HFD-induced weight gain (Figure 1B). To determine whether the reduction in body weight was associated with altered energy intake, food consumption was monitored weekly. No significant difference in average food intake was observed between the HFD and FUC groups (Figure 1C). Consistent with these findings, HFD feeding markedly increased both white adipose tissue (WAT) and brown adipose tissue (BAT) weights, whereas FUC administration significantly reduced WAT and BAT weights (Figures 1D, E), suggesting a robust protective effect against diet-induced adiposity. H&E staining further demonstrated that adipocyte hypertrophy was markedly increased in WAT of HFD-fed mice compared with CON mice (Supplementary Figure 1A). Notably, FUC administration substantially alleviated adipocyte enlargement (Figure 1F), indicating an attenuation of lipid accumulation in adipose depots. Collectively, these results demonstrate that FUC effectively mitigates HFD-induced weight gain and adipose tissue expansion in MAFLD mice.

**Figure 1:**
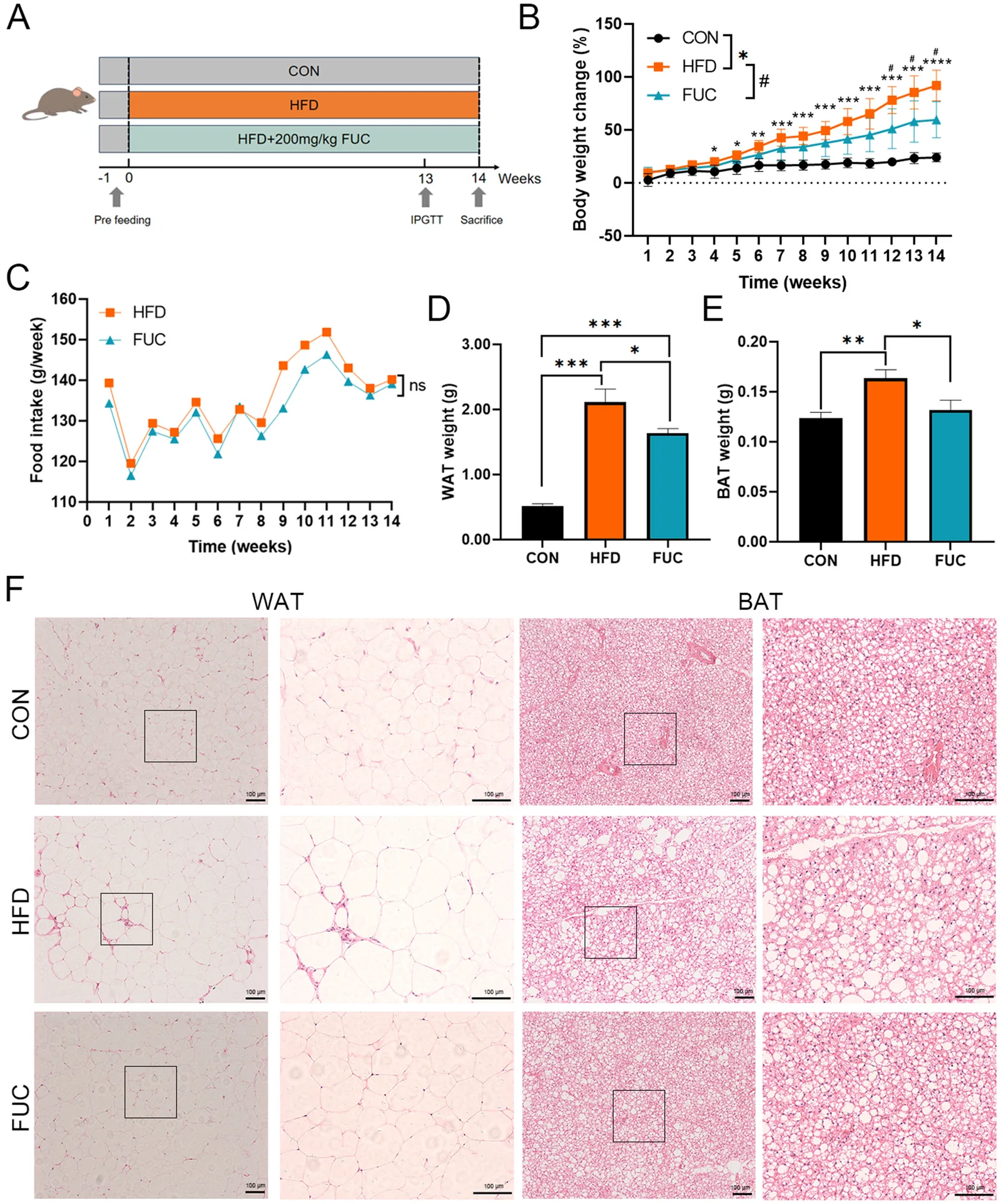
FUC reduces body weight gain and adipose tissue mass in HFD-induced MAFLD mice. **(A)** Design of animal experiments. **(B)** Body weight changes of three groups. **(C)** Changes in food intake of the three groups of mice. **(D)** Weight of WAT. **(E)** The weight of BAT. **(F)** H&E staining of WAT and BAT. Scale bar, 100 μm. Data are presented as mean ± SD (*n* = 6). *P < 0.05, **P < 0.01, ***P < 0.001; ^#^ *P* < 0.05.

### 3.2 FUC improves lipid and glucose metabolic disorders in HFD-induced MAFLD mice

Dyslipidemia, obesity, and impaired glucose tolerance are key metabolic complications of MAFLD (Accacha et al., 2025). To evaluate the effects of FUC on systemic lipid and glucose metabolism in MAFLD mice, serum TG and TC levels were dynamically monitored. Compared with the CON group, HFD feeding progressively elevated circulating TG and TC levels. In contrast, FUC administration consistently reduced both lipid parameters. Notably, TC levels were significantly lower in the FUC group at week 12 (*P* < 0.05), while TG levels were markedly reduced at week 14 compared with the HFD group, indicating that FUC effectively attenuated diet-induced hyperlipidemia (Figures 2A, B). To further assess glucose tolerance, an IPGTT was performed. HFD-fed mice exhibited significantly elevated blood glucose levels at all time points (0, 15, 30, 60, and 120 min) compared with the CON group (Figure 2C). In contrast, FUC administration significantly decreased blood glucose levels compared with the HFD group at the corresponding time points, indicating an improvement in HFD-induced hyperglycemia. Accordingly, the AUC was markedly increased in HFD-fed mice, indicating impaired glucose tolerance (Figure 2D). Consistently, both fasting blood glucose and random blood glucose levels were significantly higher in the HFD group than in the CON group. In contrast, FUC administration markedly reduced fasting and random glucose levels, suggesting improved systemic glycemic control (Figures 2E, F). Collectively, these findings indicate that concurrent FUC administration attenuated HFD-induced dyslipidemia and impaired glucose tolerance.

**Figure 2:**
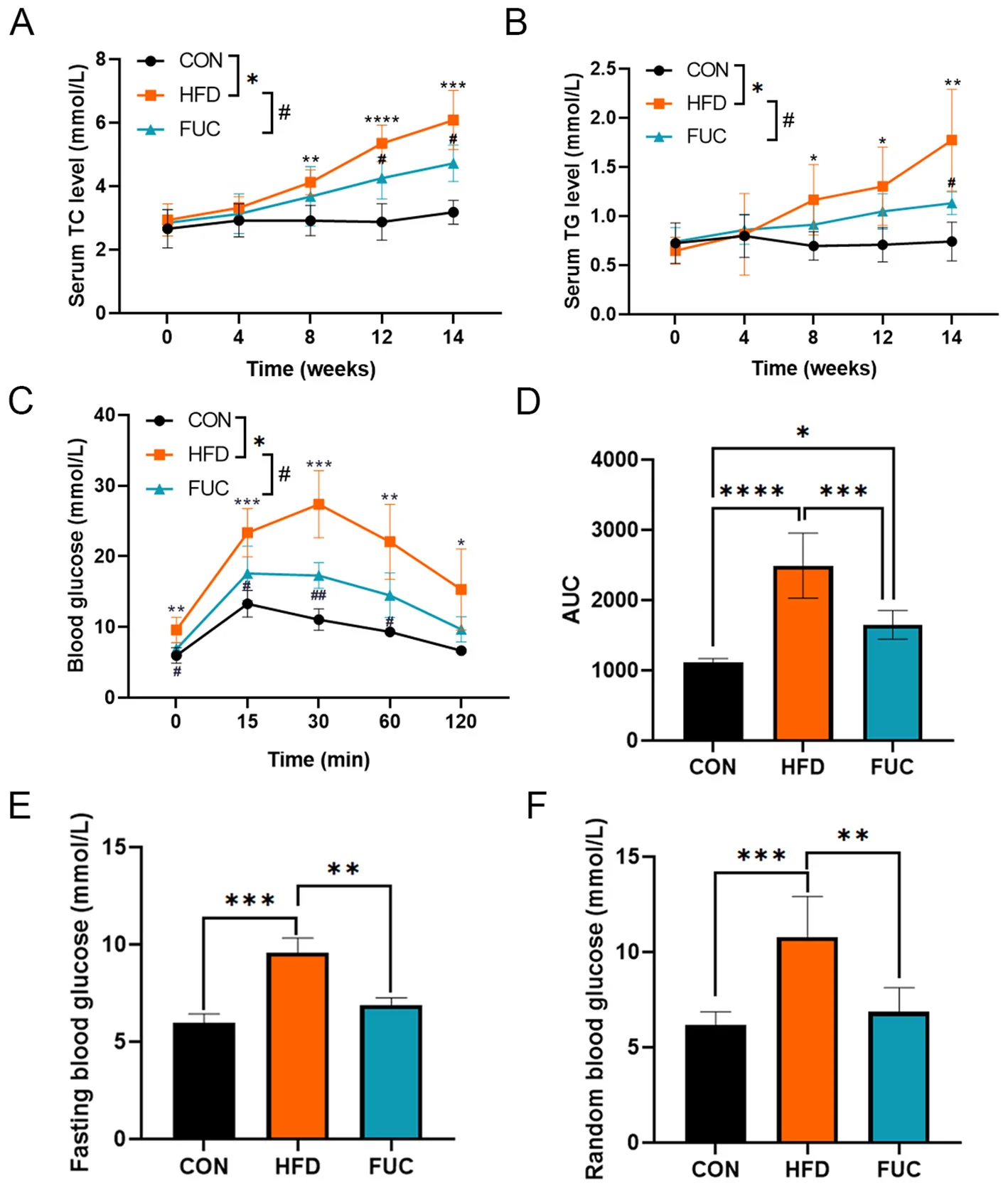
FUC improves lipid and glucose metabolic disorders in HFD-induced MAFLD mice. **(A)** Serum TC levels. **(B)** Serum TG levels. **(C)** Glucose tolerance in mice. **(D)** AUC curve of glucose tolerance. **(E)** Fasting blood glucose levels. **(F)** Random blood glucose levels. Data are presented as mean ± SD (*n* = 6). *P < 0.05, **P < 0.01, ***P < 0.001, ****P < 0.0001; ^#^ *P* < 0.05, ^##^*P* < 0.01.

### 3.3 FUC alleviates HFD-induced hepatic injury in MAFLD mice

Hepatic steatosis is a hallmark of MAFLD (Heeren and Scheja, 2021). To evaluate the effects of FUC on HFD-induced hepatic injury, serum ALT and AST levels were dynamically monitored. HFD feeding resulted in a progressive elevation of ALT and AST levels, which were significantly higher than those in the CON group from week 8 onward (*P* < 0.05) and exhibited a time-dependent increase. In contrast, FUC administration markedly attenuated the elevation of both ALT and AST levels, with a sustained protective effect observed throughout the intervention period (Figures 3A, B). Consistently, FUC significantly reduced liver weight compared with the HFD group (Figure 3C). Macroscopic examination revealed enlarged, pale, and lipid-laden livers in HFD-fed mice, whereas FUC-treated mice displayed markedly improved liver appearance (Figure 3D). Histological analysis further confirmed these findings. H&E staining demonstrated pronounced macrovesicular steatosis and hepatocyte swelling in the HFD group, whereas FUC administration substantially alleviated hepatic injury and steatosis (Figure 3D). In addition, ORO staining revealed extensive lipid droplet accumulation in HFD-fed mice, which was markedly reduced following FUC administration (Figure 3D). Quantitative analysis further showed that the ORO-positive area was significantly increased by HFD feeding and substantially reduced by FUC administration (Supplementary Figure 1B). Collectively, these results demonstrate that FUC effectively attenuates HFD-induced hepatic steatosis and suppresses lipid accumulation in MAFLD mice.

**Figure 3:**
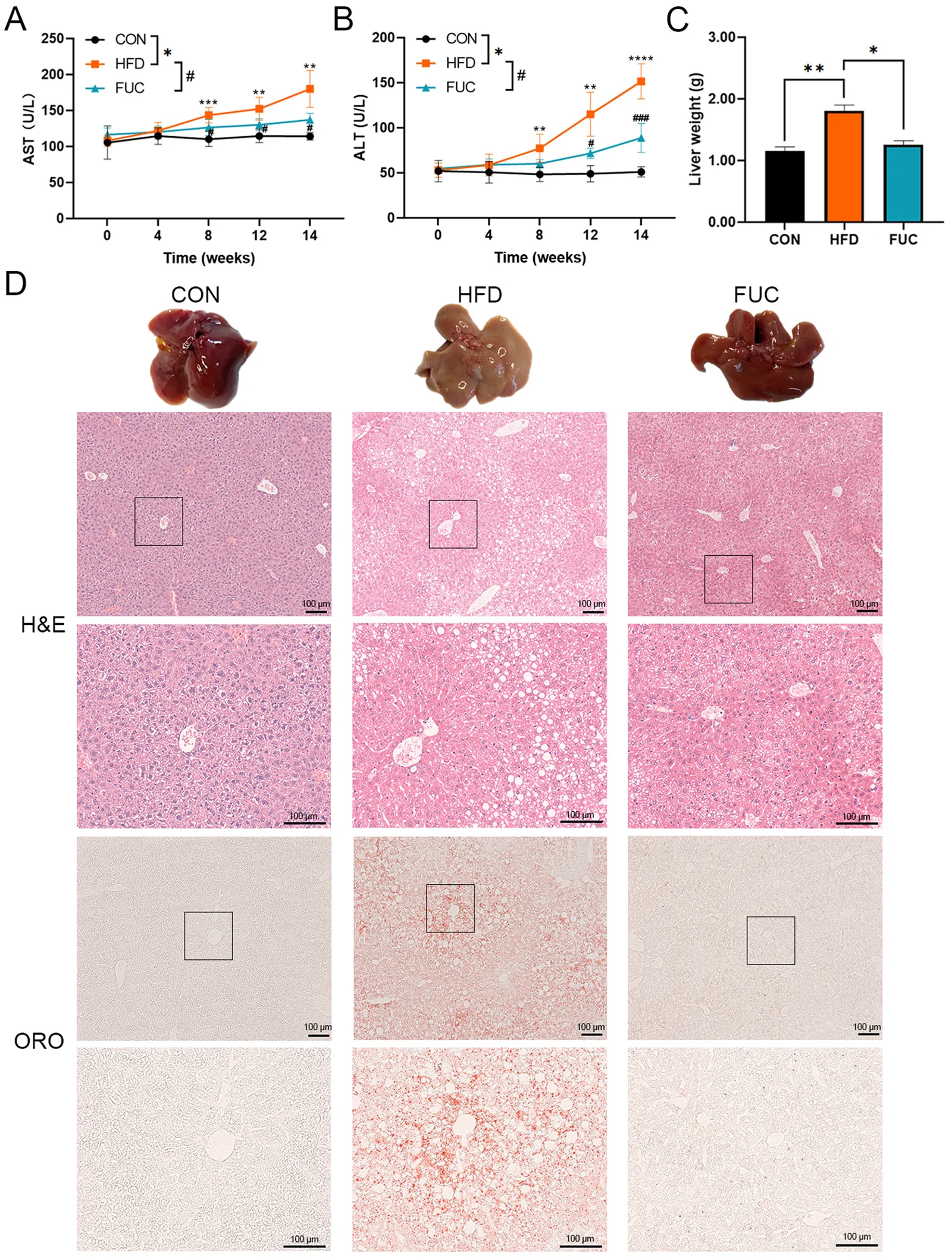
FUC alleviates HFD-induced hepatic injury in MAFLD mice. **(A)** Serum AST levels. **(B)** Serum ALT levels. **(C)** Liver weight in mice. **(D)** H&E staining and ORO staining of liver histopathological sections. Scale bar, 100 μm. Data are presented as mean ± SD (*n* = 6). *P < 0.05, **P < 0.01, ***P < 0.001, ****P < 0.0001; ^#^ *P* < 0.05, ^###^ *P* < 0.001.

### 3.4 FUC is associated with altered AMPK/PPARγ signaling in MAFLD mice

MAFLD is frequently accompanied by chronic inflammation (Cheong et al., 2025; Jang et al., 2025). To evaluate the effects of FUC on hepatic inflammation in MAFLD mice, hepatic levels of IL-6 and TNF-α were assessed. FUC administration significantly attenuated the HFD-induced increases in both IL-6 and TNF-α levels, indicating a marked anti-inflammatory effect in the liver (Figures 4A, B). To further elucidate the molecular mechanism underlying the protective effects of FUC in MAFLD, proteins related to the AMPK/PPARγ signaling axis were evaluated. Western blot analysis revealed that HFD significantly suppressed AMPK phosphorylation (p-AMPK), as evidenced by a reduced p-AMPK/AMPK ratio. In contrast, FUC administration markedly increased the p-AMPK/AMPK ratio (Figures 4C, D). Compared with the HFD group, PPARγ expression was significantly down regulated following FUC intervention (Figures 4C, E). These findings indicate that FUC administration was associated with increased hepatic AMPK phosphorylation and decreased expression levels of PPARγ, suggesting a potential involvement of AMPK/PPARγ signaling in the metabolic improvements observed in MAFLD mice.

**Figure 4:**
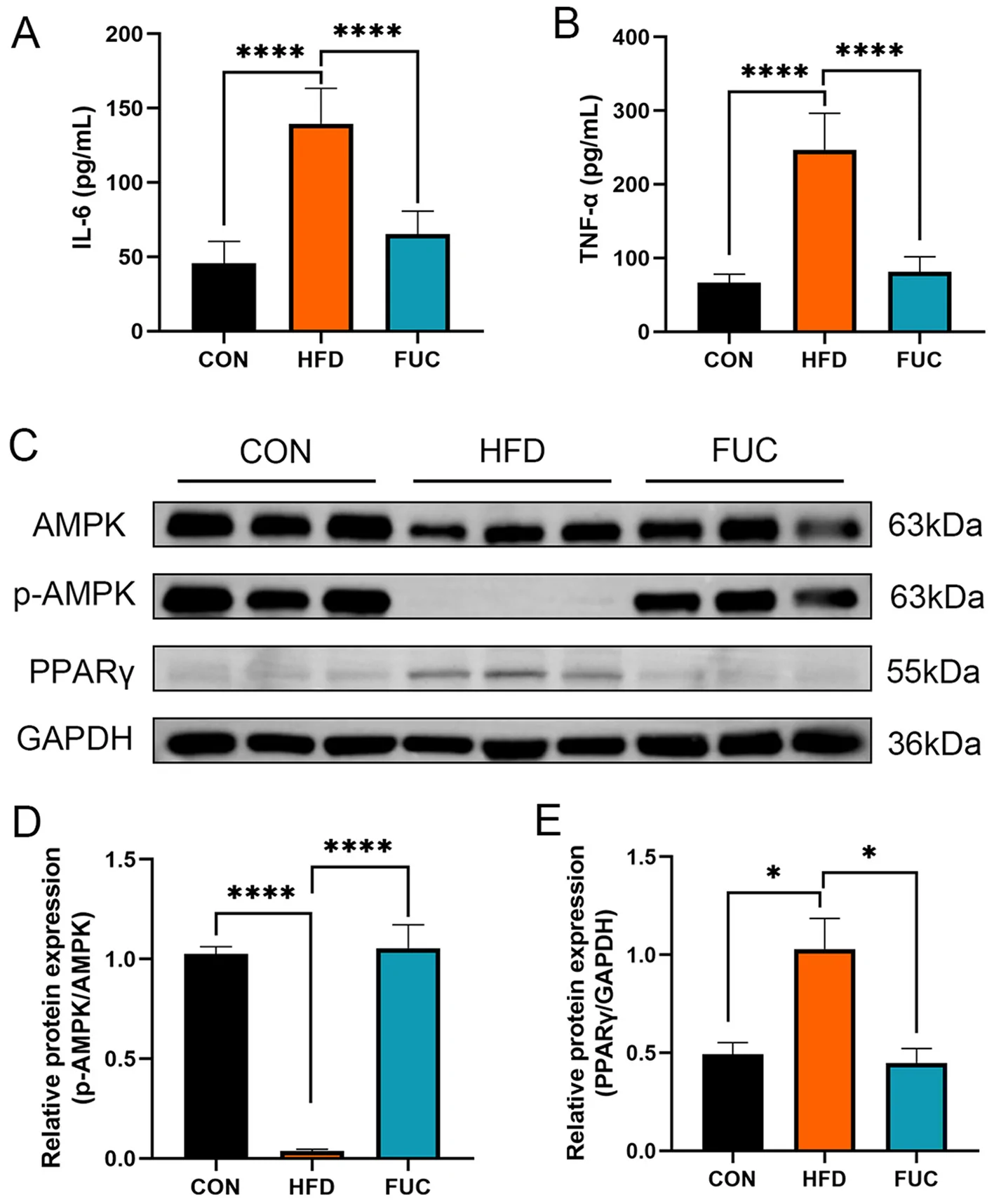
FUC is associated with altered AMPK/PPARγ signaling axis in MAFLD mice. **(A)** Liver IL-6 levels. (*n* = 6). **(B)** Liver TNF-α levels. (*n* = 6). **(C)** Representative images of Western blotting for p-AMPK, AMPK, and PPARγ in the liver. (*n* = 3). **(D)** Relative protein expression levels of p-AMPK and AMPK in the liver. (*n* = 3). **(E)** Relative protein expression levels of PPARγ in the liver (*n* = 3). Data are presented as mean ± SD. *P < 0.05, ****P < 0.0001.

### 3.5 FUC improves intestinal barrier-associated features in HFD-induced MAFLD mice

MAFLD is frequently accompanied by impaired intestinal barrier integrity (Matboli et al., 2025). HFD feeding markedly reduced colon length, whereas FUC administration significantly restored intestinal length (Figures 5A, B), indicating an improvement in gut structural integrity. H&E demonstrated that intestinal architecture in CON mice remained intact, with well-organized mucosal villi and crypt structures and no obvious inflammatory infiltration in the lamina propria. In contrast, HFD induced severe villus disruption, including shortening and fragmentation, whereas FUC administration substantially reversed these alterations (Figure 5C). AB/PAS staining revealed a marked reduction in goblet cell numbers and acidic mucin secretion in HFD-fed mice compared with CON mice. Notably, FUC administration effectively restored goblet cell abundance and mucin production (Figures 5C, D). To further characterize the molecular changes associated with intestinal barrier-related features, expression levels of tight junction and mucus-associated proteins were evaluated. RT-qPCR analysis showed that HFD significantly downregulated Occludin and MUC2 mRNA expression, whereas FUC administration reversed these changes (Figures 5H, I). Consistently, Western blot analysis results confirmed that HFD reduced Occludin and MUC2 protein levels, while FUC markedly upregulated their expression (Figures 5E–G). Collectively, these findings indicate that FUC was associated with improvements in intestinal barrier-associated structural and molecular features in HFD-induced MAFLD mice.

**Figure 5:**
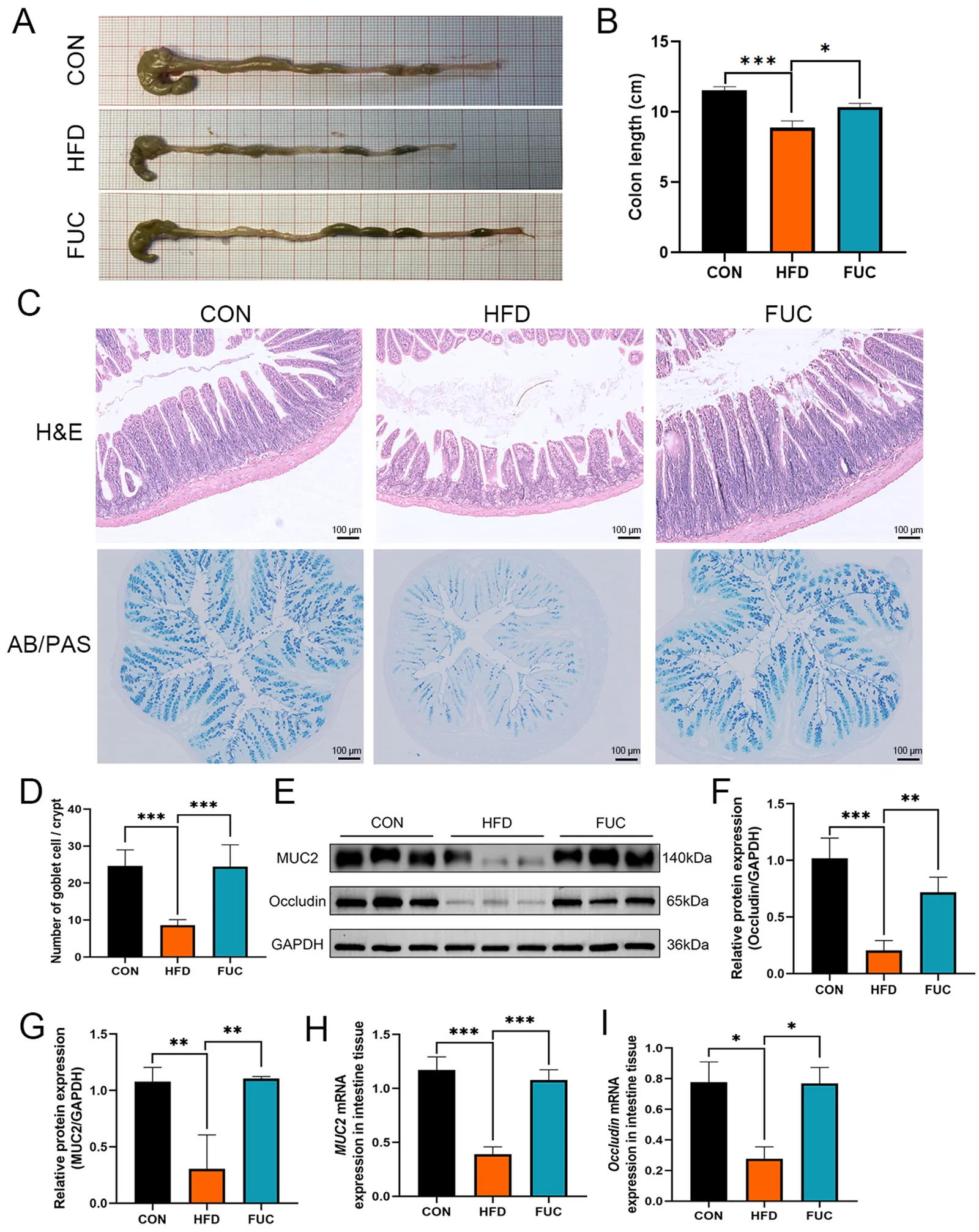
FUC improves intestinal barrier-associated features in HFD-induced MAFLD mice. **(A)** Representative images of the mice colon (*n* = 6). **(B)** Colon length (*n* = 6). **(C)** Representative H&E staining images of ileal tissues and AB/PAS staining images of colonic tissues. Scale bars, 100 μm. **(D)** Quantification of goblet cell numbers per crypt (*n* = 5). **(E)** Representative images of Western blotting for MUC2 and Occludin in colonic tissues (*n* = 3). **(F, G)** Relative protein expression levels of MUC2 and Occludin in colonic tissues (*n* = 3). **(H)** The mRNA expression levels of MUC2 in colonic tissues (*n* = 6). **(I)** The mRNA expression levels of Occludin in colonic tissues (*n* = 6). Data are presented as mean ± SD *P < 0.05, **P < 0.01, ***P < 0.001.

### 3.6 FUC is associated with remodeling of the gut microbiota in HFD-induced MAFLD mice

The gut microbiota influence hepatic physiology and disease progression through multiple mechanisms along the gut-liver axis (Wang et al., 2025). To investigate the effects of FUC on gut microbiota in MAFLD mice, 16S rRNA gene sequencing was performed. α-diversity analysis revealed that both Chao and Shannon indices were significantly reduced in the HFD group compared with the CON group, whereas FUC administration partially restored microbial richness and evenness (Figures 6A, B). PCoA analysis demonstrated a clear separation among the three groups (PERMANOVA, *P* = 0.001), indicating that FUC substantially reshaped the gut microbial community structure (Figure 6C). At the phylum level, FUC reduced the *Bacillota*/*Bacteroidota* ratio. At the genus level, FUC significantly enriched beneficial taxa, including *Akkermansia* and *Lachnospiraceae_NK4A136_group*, while reducing the abundance of *Dubosiella* (Figures 6D–I). The relative abundance of *Adlercreutzia* was also higher in the FUC group than in the HFD group (Figure 6J). Exploratory LEfSe analysis identified distinct microbial taxa associated with the three experimental groups (Figure 6K). Collectively, these findings indicate that FUC administration was associated with remodeling of the gut microbial community, including changes in the relative abundances of *Akkermansia, Adlercreutzia*, and *Dubosiella*. These taxa were therefore considered representative microbial features associated with FUC administration rather than independently validated microbial biomarkers.

**Figure 6:**
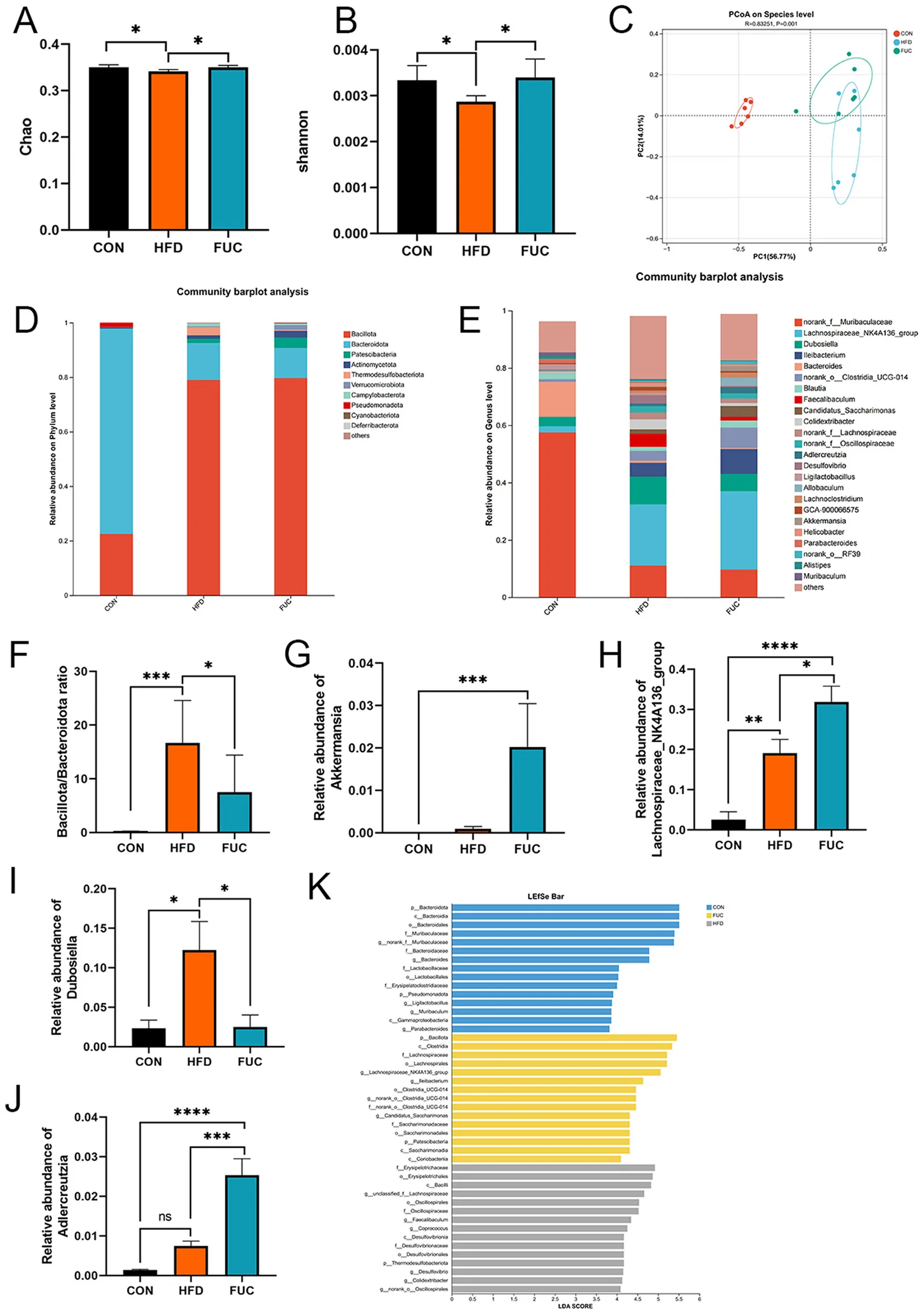
FUC is associated with remodeling of the gut microbiota in HFD-induced MAFLD mice. **(A)** Chao indices. **(B)** Shannon indices. **(C)** Bray-curtis PCoA of gut micro biota across groups (PERMANOVA, *P* = 0.001). **(D)** The relative abundance of the microbiome at the phylum level. **(E)** The relative abundance of the micro biome at the genus level. **(F)** The *Bacillota*/*Bacteroidota* ratio. Relative abundance of *Akkermansia*. **(G)**, *Lachnospiraceae_NK4A136_group* **(H)**, *Dubosiella* **(I)**, *Adlercreutzia* **(J)**, at the genus level. **(K)** Microbial markers screened based on LEfSe score (showing top 15 genera per group). Data are presented as mean ± SD (*n* = 6). *P < 0.05, **P < 0.01, ***P < 0.001, ****P < 0.0001.

### 3.7 FUC reshapes serum metabolomic profiles in HFD-induced MAFLD mice

Serum meta bolomic profiling was performed to characterize systemic metabolic alterations in MAFLD mice, as serum metabolites reflect global metabolic changes (Shao et al., 2023). PCA analysis demonstrated a clear separation among groups, indicating that FUC markedly reshaped the systemic metabolic landscape (Figure 7A). Differential metabolite analysis revealed 179 upregulated and 445 downregulated metabolites in the HFD group compared with the CON group (Figure 7B). In contrast, 34 metabolites were upregulated and 209 metabolites were downregulated in the FUC group compared with the HFD group (Figure 7C). KEGG pathway enrichment analysis of differential metabolites between the FUC and HFD groups indicated that these metabolites were primarily associated with metabolic pathways, including amino acid metabolism, carbohydrate metabolism, and lipid metabolism (Figure 7D). Further KEGG enrichment analysis revealed that multiple metabolic pathways were significantly altered by FUC, with the TCA cycle representing the most significantly enriched pathway (Figure 7E). Targeted analysis of TCA cycle–related metabolites showed that citric acid, cis-aconitic acid, L-malic acid, and fumaric acid were markedly elevated in the HFD group compared with the CON group, whereas FUC administration reversed these alterations (Figure 7F). Collectively, these findings indicate that HFD-induced MAFLD was associated with alterations in circulating TCA cycle-related metabolites, including citrate, cis-aconitate, L-malate, and fumarate. FUC administration partially reversed these metabolite alterations, suggesting an association with improved systemic metabolic homeostasis.

**Figure 7:**
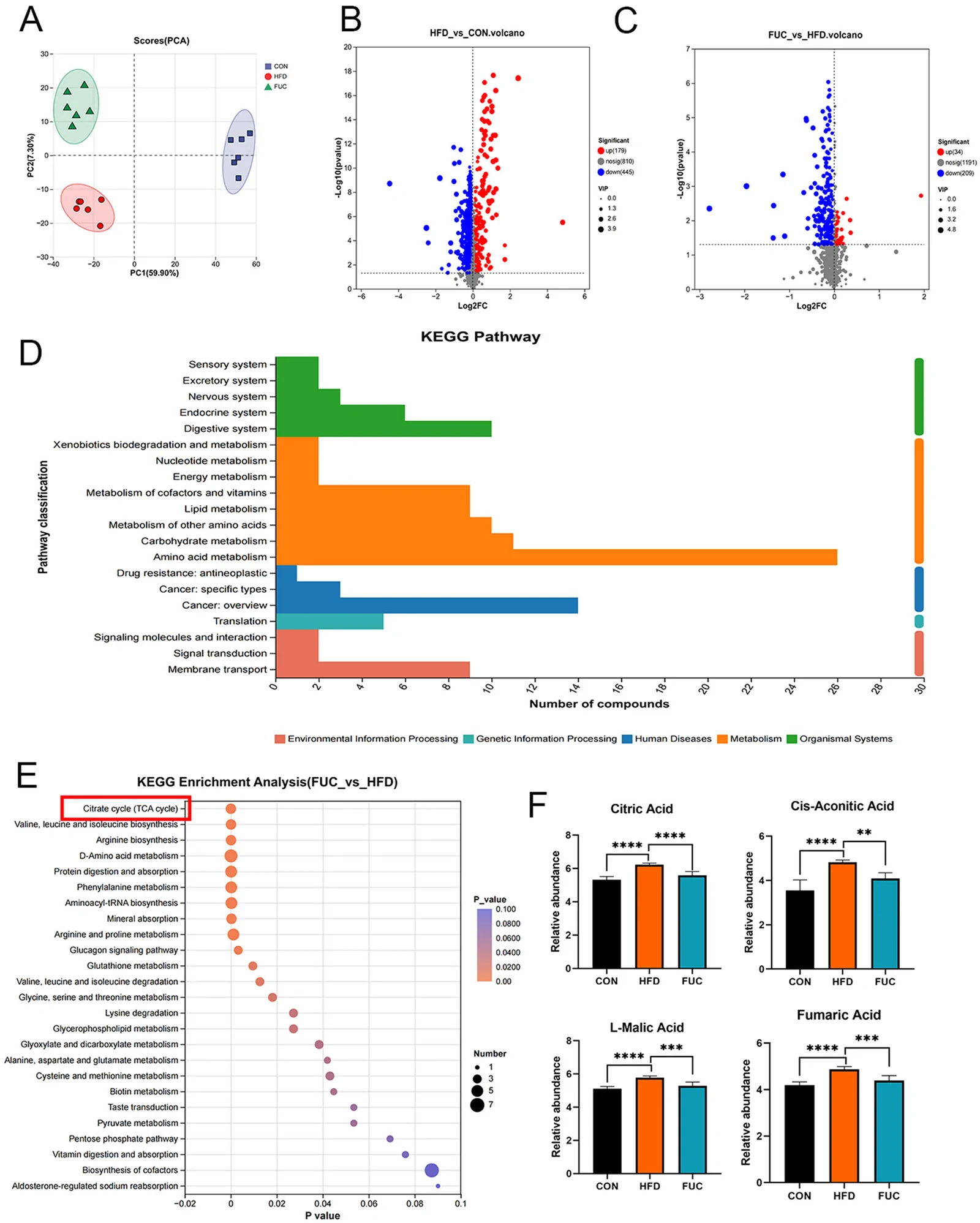
FUC reshapes serum metabolomic profiles in HFD-induced MAFLD mice. **(A)** Multivariate statistical PCA analysis. Volcano plot of differentially expressed metabolites showing the log2 ratio of metabolite expression vs. -log10 p value in HFD group compared to CON group. **(B)** and FUC group compared to HFD group. **(C, D)** KEGG pathway enrichment analysis of differential metabolites between the FUC and HFD groups. **(E)** KEGG enrichment analysis (FUC vs. HFD). **(F)** TCA cycle–related metabolites (citric acid, cis-aconitic acid, L-malic acid, and fumaric acid). Data are presented as mean ± SD (*n* = 6). **P < 0.01, ***P < 0.001, ****P < 0.0001.

### 3.8 Correlation analysis between serum metabolites, gut microbiota, and biochemical parameters

To further explore the relationships among systemic metabolism, gut microbiota, and biochemical parameters in MAFLD mice, Spearman correlation analysis was performed using representative TCA cycle metabolites, citric acid and fumaric acid. Correlation analysis with gut microbiota revealed that both citric acid and fumaric acid were positively associated with the relative abundance of *Dubosiella* (Figures 8A, B), which was increased in the HFD group and reduced following FUC administration. Both metabolites were also positively associated with serum TC and TG levels at week 14 (Figures 8C–F). Collectively, these findings suggest that citric acid and fumaric acid may serve as potential metabolic biomarkers of lipid metabolic dysregulation in MAFLD. Furthermore, these correlations suggest potential associations among gut microbial composition, circulating TCA cycle-related metabolites, and metabolic phenotypes in MAFLD mice. FUC administration was associated with partial normalization of these metabolic–microbial relationships.

**Figure 8:**
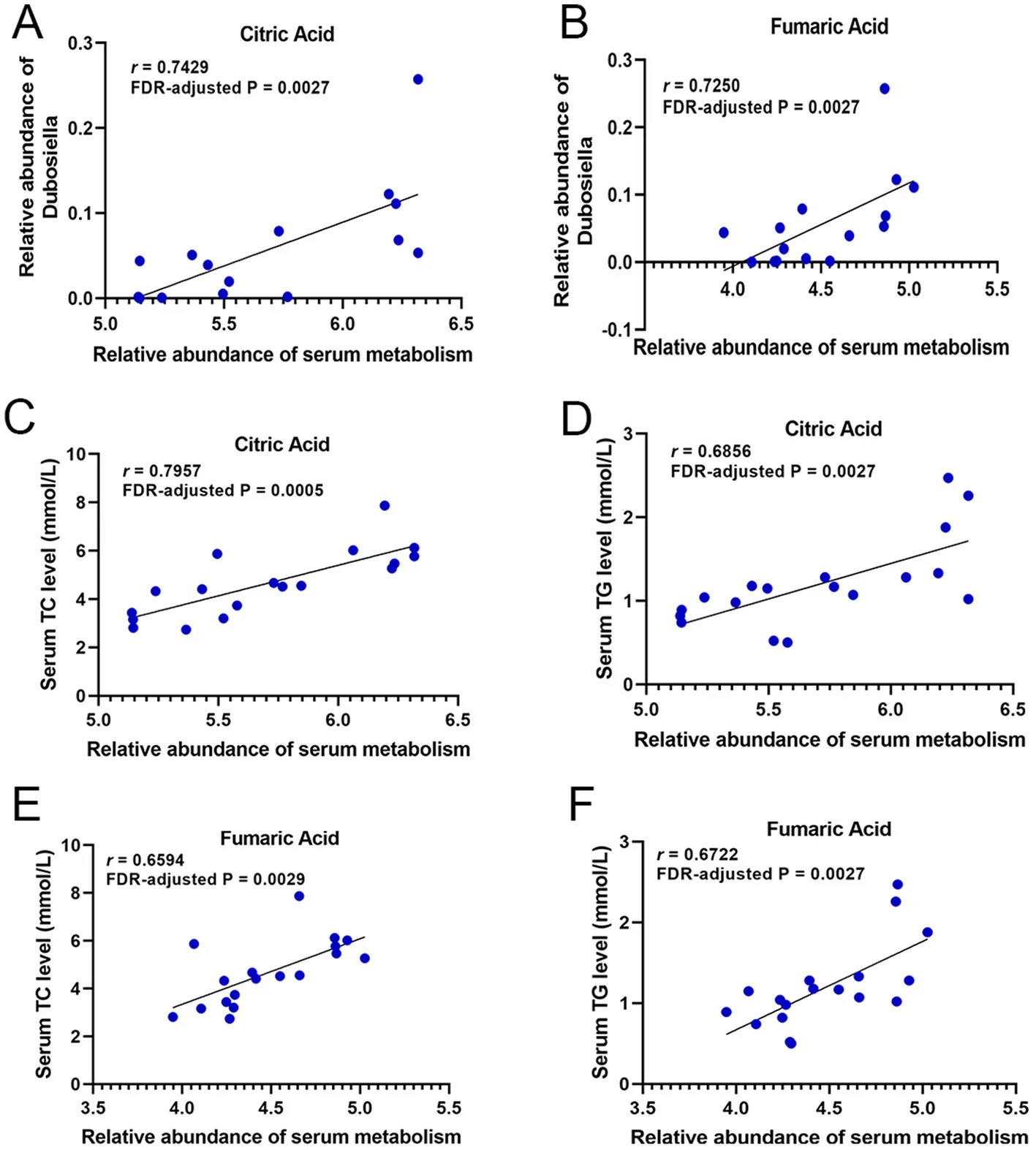
Correlation analysis between serum metabolites, gut microbiota, and biochemical parameters. **(A)** Spearman correlation curves between citric acid and *Dubosiella*. **(B)** Spearman correlation curves between fumaric acid and *Dubosiella*. **(C-F)** Spearman correlation curves between citric acid/fumaric acid and biochemical parameters (TC and TG).

## 4 Discussion

MAFLD has emerged as one of the most prevalent chronic liver diseases worldwide, with a complex pathogenesis involving dysregulated lipid metabolism, impaired glucose homeostasis, chronic inflammation, and gut micro biota imbalance (Badmus et al., 2022; Ji et al., 2023; Li et al., 2026). In this study, we systematically evaluated the effects of FUC in an HFD-induced MAFLD mouse model. Our results demonstrate that FUC significantly attenuates weight gain, reduces lipid accumulation, improves hepatic injury, and attenuates impaired glucose tolerance. More importantly, FUC administration was associated with improvements in intestinal barrier-associated features, changes in gut microbiota composition, and alterations in the AMPK/PPARγ signaling axis. Together, these findings highlight the gut–metabolism–liver axis as a relevant context for understanding the effects of FUC in HFD-induced MAFLD.

Given its high molecular weight and sulfated polysaccharide structure, FUC exhibits limited systemic bioavailability. Therefore, its biological effects are increasingly considered to be mediated through gut micro biota-dependent mechanisms (Sun et al., 2025). Gut bacteria can degrade FUC into bioactive oligosaccharides and metabolites, which may further regulate host energy metabolism (Hamouda et al., 2025). In the present study, the metabolic benefits of FUC were closely associated with alterations in gut microbial composition, including enrichment of beneficial taxa and restoration of microbial diversity. These findings support the concept that the gut micro biota acts as a critical mediator of FUC activity rather than merely metabolic improvement.

Obesity is a major driver of MAFLD progression. Chronic HFD feeding disrupts energy homeostasis, leading to excessive lipid influx into the liver and ectopic fat deposition. In the present study, FUC significantly attenuated body weight gain and reduced adipose tissue mass without a detectable change in food intake. These findings suggest that the effects of FUC on body weight were not accompanied by a significant alteration in food intake. However, whether these effects involve changes in energy expenditure or lipid utilization remains to be determined. These findings are consistent with previous reports showing that FUC promotes fatty acid oxidation, enhances energy expenditure, and induces adipose tissue browning, further supporting its anti-obesity potential (Chu et al., 2025; Yin et al., 2024). Hepatic lipid metabolic dysregulation represents a central pathological feature of MAFLD (Willis et al., 2021). Under physiological conditions, hepatic lipid homeostasis is maintained through a dynamic balance among fatty acid uptake, synthesis, oxidation, and export. When this balance is disrupted, triglycerides accumulate in hepatocytes, leading to steatosis (Miao et al., 2024). In this study, FUC significantly reduced serum ALT, AST, TC, and TG levels, and alleviated hepatic steatosis and lipid deposition, as evidenced by histological analyses. These results indicate that FUC not only improves systemic metabolic disorders but also directly attenuates hepatic lipid accumulation and tissue injury.

AMPK serves as a central energy sensor and metabolic regulator and is considered a key therapeutic target for MAFLD (Alshehade et al., 2022). Upon energy stress, AMPK is activated via phosphorylation, subsequently inhibiting lipogenesis while promoting fatty acid β-oxidation to restore energy homeostasis (Sethi et al., 2025). In this study, HFD suppressed AMPK phosphorylation, whereas FUC increased the hepatic p-AMPK/AMPK ratio. Meanwhile, FUC administration reduced hepatic PPARγ expression. Given that AMPK and PPARγ are both involved in the regulation of hepatic lipid metabolism, these coordinated changes may reflect an altered AMPK/PPARγ signaling state associated with the metabolic benefits of FUC. Collectively, these findings suggest that altered AMPK/PPARγ signaling may be associated with the hepato protective effects of FUC. Nevertheless, because pharmacological inhibition or genetic manipulation of AMPK/PPARγ was not performed, the causal contribution of this signaling axis to the effects of FUC remains to be established.

Inflammation is a central driver of MAFLD, closely interacting with dysregulated lipid metabolism and insulin resistance to accelerate hepatic injury (Filipovic et al., 2023; Zhang et al., 2021). Accumulating evidence indicates that MAFLD is not merely a metabolic disorder but a chronic low-grade inflammatory disease characterized by sustained activation of innate immune responses (Tauil et al., 2024). In this context, pro-inflammatory cytokines, including IL-6 and TNF-α, play pivotal roles in promoting hepatocellular stress, immune cell infiltration, and fibrogenic signaling (Liu et al., 2025; Wang et al., 2026). In the present study, FUC markedly reduced hepatic IL-6 and TNF-α levels in HFD-fed mice, indicating that it exerts potent anti-inflammatory effects in addition to metabolic regulation. Mechanistically, excessive hepatic lipid accumulation induces lipo toxic stress, mitochondrial dysfunction, and reactive oxygen species (ROS) production, thereby activating inflammatory signaling cascades such as NF-κB and JNK. These pathways further amplify cytokine production and sustain chronic hepatic inflammation (Clare et al., 2022; Stachowicz et al., 2024). In this study, FUC significantly alleviated hepatic steatosis and improved lipid metabolic parameters, suggesting that its anti-inflammatory effects may, at least in part, result from reduced lipid overload and restoration of metabolic homeostasis. Notably, activation of the AMPK/PPARγ signaling axis may also directly contribute to inflammation resolution. AMPK activation has been shown to inhibit NF-κB signaling and suppress pro-inflammatory gene transcription (Marques et al., 2023; Peng et al., 2022; Sheng et al., 2025). Thus, the increased hepatic AMPK phosphorylation observed following FUC administration may represent one potential link between metabolic improvement and the attenuation of hepatic inflammation.

In addition, gut-derived inflammatory signaling has emerged as a key contributor to MAFLD progression (Aghara et al., 2025). Disruption of intestinal barrier integrity facilitates translocation of microbial products, such as lipopolysaccharide (LPS), into the portal circulation, thereby triggering hepatic immune activation and sustaining inflammation (Zhou et al., 2025). The gut-liver axis has therefore become a central framework in MAFLD research (Qi et al., 2025; Yang et al., 2023), whereby gut micro biota regulate hepatic metabolism through bile acid signaling, short-chain fatty acid production, intestinal barrier maintenance, and inflammatory modulation (Hu et al., 2025). In this study, HFD feeding was associated with alterations in intestinal barrier-associated structural and molecular features, including reduced expression of tight junction proteins and decreased mucin production, whereas FUC administration partially reversed these changes. Concomitantly, HFD-induced gut dysbiosis was accompanied by an increased *Bacillota/Bacteroidota* ratio, whereas FUC administration significantly reduced this ratio. Although shifts in the Bacillota/Bacteroidota ratio have been associated with metabolic disorders (Vázquez-Bolea et al., 2026), this ratio alone should not be considered a definitive indicator of metabolic health. In the present study, FUC administration significantly reduced the *Bacillota*/*Bacteroidota* ratio, which may represent one component of the broader gut microbial remodeling associated with FUC administration. In parallel, FUC administration was associated with changes in the relative abundances of several microbial genera, including increased *Akkermansia* and *Adlercreutzia* and decreased *Dubosiella*. These genera have previously been associated with metabolic phenotypes in different experimental or clinical contexts (Asghari et al., 2025). However, the present study was not designed to establish the independent functional roles of these taxa in mediating the effects of FUC. Moreover, the differential abundance analysis was based on exploratory LEfSe screening rather than independent confirmation using compositionality-aware methods such as ANCOM-BC2 or MaAsLin2. Therefore, these microbial changes should be interpreted as FUC-associated features of the gut microbiome rather than definitive microbial biomarkers or causal mediators. Interestingly, the latest research shows that *Akkermansia muciniphila* administration reduces hepatic lipid accumulation and enhances liver regeneration in MAFLD models (Wu et al., 2023). *Adlercreutzia* has been implicated in host lipid metabolism regulation, with increased abundance consistently associated with reduced hepatic steatosis and improved serum lipid profiles (Duan et al., 2025). Its potential mechanisms include modulation of bile acid metabolism, suppression of chronic inflammation, and interaction with other short-chain fatty acid (SCFA)–producing microbes (Gómez-García et al., 2025; Kumar et al., 2025). In the present study, we did not directly measure bile acids, SCFAs, or other metabolites specifically linked to these taxa. Therefore, the functional consequences of the FUC-associated enrichment of *Adlercreutzia* and other microbial taxa remain to be determined. Our findings suggest that FUC may act as a microbiota-modulating nutritional intervention associated with coordinated changes in gut microbial composition, intestinal barrier-associated features, and systemic metabolic regulation. The metabolic benefits of FUC were accompanied by marked changes in gut microbial composition, including the enrichment of taxa associated with metabolic health and partial restoration of microbial diversity. However, given that FUC also reduced body weight gain and adipose tissue accumulation, and that metabolic status can itself influence the gut micro biota, these microbial changes may reflect either direct effects of FUC or secondary consequences of improved metabolic health. Therefore, our findings indicate an association between FUC administration and gut microbiota remodeling but do not establish a causal role for the microbiota in mediating the metabolic benefits of FUC.

To further elucidate functional mechanisms associated with FUC administration, untargeted serum meta bolomic profiling was performed. FUC significantly altered serum metabolic profiles, with TCA cycle–related pathways identified as the most prominently affected (Hu et al., 2025). Mitochondrial dysfunction is a hallmark of MAFLD and plays a central role in disease progression (Zhao et al., 2023). Excessive lipid accumulation enhances fatty acid β-oxidation flux, increasing acetyl-CoA input into the TCA cycle. However, sustained lipid overload impairs mitochondrial oxidative phosphorylation, resulting in incomplete substrate oxidation and accumulation of TCA intermediates. Among these metabolites, citric acid represents a key metabolic hub linking mitochondrial energy production and *de novo* lipogenesis. Its accumulation may reflect mitochondrial metabolic stress and enhanced lipid synthesis (Udoh et al., 2025). Fumaric acid has recently been recognized not only as a TCA intermediate but also as an immune metabolic regulator associated with oxidative stress and inflammatory signaling (Xaviera et al., 2024). In this study, meta bolomic analysis further revealed substantial changes in serum metabolic profiles following FUC administration, with the TCA cycle identified as one of the most significantly enriched pathways. In particular, circulating citrate, cis-aconitate, L-malate, and fumarate were elevated in HFD-fed mice and partially reversed by FUC administration. These findings suggest that FUC is associated with changes in systemic TCA cycle-related metabolism. However, circulating metabolite concentrations do not directly reflect mitochondrial TCA-cycle flux or mitochondrial functional activity. Therefore, the observed metabolic changes should be interpreted as alterations in circulating metabolic profiles rather than evidence of restored mitochondrial TCA-cycle activity. Correlation analyses further supported the functional association among gut micro biota, serum metabolites, and biochemical parameters, highlighting a coordinated gut–metabolism interaction in MAFLD progression.

This study has several limitations. First, FUC was administered concurrently with HFD exposure, representing a preventive rather than therapeutic intervention. Thus, our findings indicate attenuation of HFD-induced metabolic and hepatic abnormalities but do not establish efficacy against established MAFLD. Second, only a single FUC dose (200 mg/kg/day) was tested, and a standard chow + FUC group was not included; therefore, dose-dependent effects and effects in the absence of HFD exposure remain unclear. Third, only male mice were used, limiting the generalizability of the findings to females. Fourth, although FUC administration was associated with substantial gut microbial remodeling, these changes may be influenced by the accompanying reduction in body-weight gain and improved metabolic status. Thus, the present study cannot distinguish direct effects of FUC on the micro biota from secondary changes associated with metabolic improvement. Fifth, FUC reduced body-weight gain without significantly affecting food intake; however, energy expenditure, nutrient absorption, and fecal energy or lipid content was not assessed, and the mechanisms underlying this phenotype therefore remain unclear. Sixth, improvements in colonic morphology and MUC2 and Occludin expression represent barrier-associated structural and molecular changes rather than direct evidence of restored intestinal permeability. Finally, microbiome and meta bolomic analyses identified associations but did not establish causality. In particular, micro biota depletion, fecal micro biota transplantation, or germ-free animal experiments were not performed to determine whether the FUC-associated microbial changes are required for or sufficient to reproduce the metabolic benefits of FUC. Micro biota-derived metabolites, including SCFAs and bile acids, were not directly quantified. Therefore, the specific microbial or metabolic mediators of the observed effects remain to be determined.

## 5 Conclusion

In conclusion, FUC supplementation alleviated HFD-induced MAFLD and was associated with remodeling of the gut microbial community, improvements in intestinal barrier-associated structural and molecular features, and alterations in microbiota-associated metabolic profiles. FUC administration was also associated with altered hepatic AMPK/PPARγ signaling, suggesting a potential link between these alterations and the metabolic benefits of FUC. Although these findings support a potential role of gut microbiota remodeling and AMPK/PPARγ signaling in the effects of FUC, their causal contributions remain to be established. Overall, our findings highlight the potential of marine-derived polysaccharides as nutritional interventions for metabolic disorders.

## Data Availability

The raw 16S rRNA gene sequence reads generated in this study have been deposited in the NCBI Sequence Read Archive (SRA) under Bio Project accession number PRJNA1493819. The metabolomics datasets generated in this study have been deposited in the National Omics Data Encyclopedia (NODE) of the National Genomics Data Center (NGDC) under accession number OMIX018539.
