# Supplementary material for: Fucoidan attenuates metabolic dysfunction-associated fatty liver disease in mice and is associated with gut microbiota remodeling and AMPK/PPARγ signaling
Source: Front Microbiol. 2026 Oct 27;17:1936901. doi: 10.3389/fmicb.2026.1936901 (PMC13747855; doi:10.3389/fmicb.2026.1936901)
Supplement: Supplementary file 1 [file Supplementary_File_1.docx]

**Supplementary Figure**

**Supplementary Figure 1.** Quantitative histological analysis of white adipose tissue adipocyte size and hepatic lipid accumulation in HFD-induced MAFLD mice. (A) Average area of adipocytes in WAT. (B) Quantification of Oil red O (ORO) positive area per field.Data are presented as mean ± SD (n = 5). ****P* < 0.001, *****P* < 0.0001
